# Lack of Association Between *CCR3 rs6441961* T>C Polymorphism and Celiac Disease in Punjabi Pakistani Population

**DOI:** 10.34172/mejdd.3265

**Published:** 2026-01-31

**Authors:** Ansar Ali, Waqas Ahmed Khan, Misbah Hussain, Sumaira Zulfiqar, Amna Fiaz, Muhammad Adnan Masood

**Affiliations:** ^1^Department of Biotechnology, Faculty of Sciences, University of Sargodha, Sargodha, Pakistan; ^2^Department of Medicine, Niazi Medical & Dental College Sargodha, Pakistan

**Keywords:** *rs6441961*, Tetra-ARMS-PCR, Celiac disease, Punjabi Pakistani population, Polymorphism

## Abstract

**Introduction:**

Celiac disease (CD) is an autoimmune disease characterized by gluten intolerance. *CCR3 rs6441961* T>C polymorphism is widely studied in European populations, while Asian populations are the least studied for the *rs6441961*-associated CD risk.

**Methods:**

To achieve the objectives of the current study, a tetra-primer amplification refractory mutation system polymerase chain reaction (tetra-ARMS-PCR) genotyping assay was developed for genotyping of *CCR3 rs6441961* T>C polymorphism.

**Results:**

The current study explored the association of *rs6441961* with CD in the Punjabi Pakistani population and revealed that the TT and TC genotypes were prevalent in healthy controls and the patient group, while the CC genotype was only predominant in the patient group (25.8% vs. 18.2%; χ2=0.74, *P*=0.69).

**Conclusion:**

Multinomial regression analysis and Chi-square test indicate that the risk of CD in the Punjabi Pakistani population is not influenced by *CCR3 rs6441961* T>C polymorphism.

## Introduction

 Celiac disease (CD) is an autoimmune disease affecting approximately three million people worldwide.^1^ Patients with CD are sensitive to gluten (a protein found in wheat, rye, and barley), which can trigger an immune response, leading to intestinal cell damage, malabsorption of essential nutrients, diarrhea, fatigue, weight loss, and many other symptoms. The prevalence of CD is not the same in all countries. It is more prevalent in European countries as compared with the Asia-Pacific region. These variations could be due to the difference in genetic factors and per-capita wheat consumption.^2,3^ Clinical manifestations of CD are wide-ranging, from intestinal symptoms to non-intestinal symptoms,^4^ however, most of the patients exhibit intestinal problems.^5^ Usually, the diagnosis of CD is based on the titer of IgA anti-transglutaminase antibodies (IgA anti-tTG) and endoscopic findings.^6,7^ Confocal endomicroscopy can also be used for the diagnosis of CD.^8^ Saadah and colleagues ^6^ also suggested that serological assessment of the deamidated gliadin peptide of IgG can be used in CD diagnosis instead of measuring the IgA anti-tTG.

 In children, CD can influence growth and development; however, in adults, these side effects are less pronounced. CD is of major concern among all age groups owing to the unavailability of any cure/therapy for this disease. Individuals who are genetically at risk and/or whose immune system is sensitive to gluten can only manage the symptoms by strictly following a gluten-free diet.^9^ Although a gluten-free diet successfully manages CD and helps in intestinal healing, some patients do not respond to this dietary intervention and develop CD-related complications like intestinal adenocarcinoma, T-cell lymphoma, and refractory sprue.^10^ Several environmental and genetic factors contribute to the etiology of CD.^11^ Approximately 40% of the patients with CD carry human leukocyte antigen (HLA) class II haplotype DQ2 or DQ8.^12^ Studies have shown that HLA alter the intestinal microbiome composition and that dysbiosis triggers gluten sensitivity.^11^ Genome-wide studies have also identified 39 non-HLA genes that are linked with CD risk.^5,13^ Among these, *CCR3* is an important non-HLA gene encoding C-C chemokine receptor type 3 (CCR3 receptor), which is abundantly expressed on immune cells involved in allergic reactions like mast cells, eosinophils, basophils, and TH2 cell subsets.^14^ CCR3 receptor plays a vital role in basal trafficking of eosinophils to intestinal mucosa.^15^ CCR3 receptor and its respective chemokines have been implicated in several diseases like T-cell lymphoma, cutaneous lupus erythematosus.^16,17^ CCR3 receptor also acts as co-receptor for HIV-1 and help it in cell entry.^18^ CCR3 plays a key role in intestinal immune responses, and genetic variants in this gene have been implicated in chronic inflammatory and autoimmune disorders, including CD. Studies have identified that *CCR3* rs6441961 T > C increases the susceptibility for CD.^14,19,20,21^ This polymorphism is located on the 3p21 chromosomal region, which contains a gene cluster of chemokine receptors. *CCR3* also belongs to the chemokine receptor gene cluster, and this is the nearest gene located only 44 kb from *rs6441961* T > C.^22^ Although *CCR3 rs6441961* T > C has been reported in association studies across European and other populations, data from South Asian groups are limited. Given the ethnic differences in allele frequencies and disease risk, studying this variant in the Punjabi Pakistani population can provide important insights into its population-specific relevance, and assessing genetic risk factors can help in early diagnosis of CD, which can prevent long-term complications and reduce patient suffering.

 Furthermore, European countries are economically stable and commonly employ high-throughput advanced techniques like GWAS (genome-wide association studies), WES (whole exome sequencing), and TaqMan genotyping assay for genotyping of *rs6441961*.^23,24,25^ Although these techniques are quite specific and sensitive, they are costly; therefore, they cannot be used for screening of larger populations in resource-limited settings like Pakistan. Thus, less expensive and specific genotyping assays should be developed to conduct large-scale replicative studies in developing countries. One such technique is tetra-ARMS-PCR, which is simple, rapid, and cost-effective. The main objective of this study was to design a tetra-ARMS-PCR genotyping assay for genotyping of *CCR3 rs6441961* T > C and find its association with CD in the Pakistani Punjabi population.

## Materials and Methods

###  Study Population

 A total of 90 individuals from Sargodha (a city in Punjab, Pakistan) and its peripheral areas were enrolled for this study and divided into two groups: disease group (N = 35) and healthy controls (N = 55). Diseased subjects included both males and females with an age of > 10 years. CD was diagnosed by an expert gastroenterologist based on gluten sensitivity and gastrointestinal problems. IgA anti-transglutaminase (IGA anti-tTG) antibodies test, as well as endoscopy (extension of the crypts, partial to complete villous atrophy), was used to confirm the disease diagnosis. All the subjects with irritable bowel syndrome and other gastrointestinal problems were excluded.

 Healthy controls had no gastrointestinal disease. They were also screened by a gastroenterologist for subclinical gluten sensitivity, and subjects without subclinical CD were included in the control group.

 A well-informed written consent was obtained from all enrolled subjects or their guardians prior to sample collection. This study was approved by the Institutional Ethical Committee, University of Sargodha, Sargodha, Punjab, Pakistan. All the methods and procedures carried out in the current study were in accordance with the Declaration of Helsinki.

###  Blood Sample Collection & DNA Extraction 

 After obtaining informed consent, 3 mL venous blood sample was collected aseptically from all enrolled subjects by an expert phlebotomist. Genomic DNA was extracted from leukocytes by using the phenol-chloroform-isoamyl alcohol DNA extraction method elaborated by Yousaf and colleagues.^26^ DNA quality was assessed by agarose gel electrophoresis (0.8% agarose gel, 80 volts, 30 minutes) followed by visualization under UV light. Good quality DNA samples were kept on -20 °C until further analysis. However, samples with low quality or quantity of DNA were subjected to another round of DNA extraction.

###  Genetic Analysis

 To achieve the objectives of the current study, a tetra-primer amplification refractory mutation system polymerase chain reaction (tetra-ARMS-PCR) genotyping assay was developed for genotyping of *CCR3 rs6441961* T > C polymorphism. Afterwards, this in-house-developed tetra-ARMS-PCR genotyping assay was applied to genotype all recruited subjects, and the association between the aforementioned polymorphism and celiac disease was assessed using various statistical tests.

###  Method Development

 Primers for genotyping of *CCR3 rs6441961* T > C polymorphism by tetra-ARMS-PCR were design by using Primer1 software which is an online available tool (http://primer1.soton.ac.uk/primer1.html). All the primers were analyzed for various critical parameters like melting temperature, heterodimer, hairpin loop, homodimer, and GC content by using OligoAnalyzer (https://sg.idtdna.com/calc/analyzer). To assess the specific amplification of primers, an in-silico PCR was performed for rs6441961 T > C by using the in-silico PCR tool available on UCSC genome browser (https://genome.ucsc.edu/cgi-bin/hgPcr). Just like conventional PCR, tetra-ARMS-PCR does not require any additional expensive reagents or infrastructure, but in tetra-ARMS-PCR, two pairs of primers are used instead of one. The outer pair of primers, which are non-allele specific, and the inner pair of primers, which are allele specific. In an allele-specific primer at the 3’ end, the polymorphic nucleotide is present. The sensitivity and specificity of the allele-specific primer are enhanced by deliberately creating a mismatch from the 3’ end at the 3rd position (a.k.a. -3 position). Furthermore, to enhance the visibility and resolution of PCR products on agarose gel, the minimum product size was kept more than 200 bp, with a difference of at least 75 bp between allelic bands. Details of *CCR3 rs6441961* T > C primers are presented in Table 1.

**Table 1:** Tetra-ARMS-PCR primer information for genotyping of *CCR3 rs6441961* T > C

**No.**	**Primer name**	**Primer sequence (5’ to 3’)**	**Tm (**°**C)**	**Product size**
1	Forward outer	GATGATGACACCACTAGCCAACC	58	Control = 496 bp T allele = 236 bp C allele = 316 bp
2	Reverse inner	CAGGTGCTGGAGATGTTATCAAGGATA	58	
3	Forward inner	TTGAGCAATTATTTCCACTTGGTTATATGC	56	
4	Reverse outer	ATTCAGGAACAAAATGGAGACAGTCTTG	57	

 Optimized reaction mixture for successful genotyping of *CCR3 rs6441961* T > C was prepared in 200 µL PCR tube by adding 3 μL of 10X PCR buffer, 2 μL of 2mM dNTPs, 2 μL of 25mM MgCl_2_, 2 μL of DNA (100–200 ng/µL), 0.3 μL of Taq polymerase (5U/µL), 0.5 μL of both forward outer and reverse outer primer (10 pmol/µL) and 1μL of both forward inner and reverse inner primer. Finally, 12.7 µL sterilized deionized water was added to make up the total volume of 25 µL.

 Tetra-ARMS-PCR conditions for the genotyping of *CCR3 rs6441961* T > C were: 3 minutes initial denaturation at 95°C, which was followed by 35 cycles comprises of 30 seconds denaturation at 95°C, 45 seconds annealing at 56.9°C, 1 minute extension at 72°C, followed by final extension of 10 minutes at 72°C in T100TM thermal cycler (Bio-Rad Laboratories, Inc. Berkeley, CA, USA).

 2% Agarose gel was used to visualize the amplified PCR product, in which an intercalating dye (ethidium bromide) was added. A 50 bp DNA ladder was also run along with the PCR product to estimate the size of the amplified fragments. Gel Doc TM EZ System (Bio-Rad Laboratories, Inc.) was used to visualize the gel under UV light. Tetra-ARMS-PCR analysis of *CCR3 rs6441961* T > C revealed three bands: a control band at 496 bp, a C allele band at 316 bp, and a T allele band of 236 bp was obtained (Figure 1).

**Figure 1:**
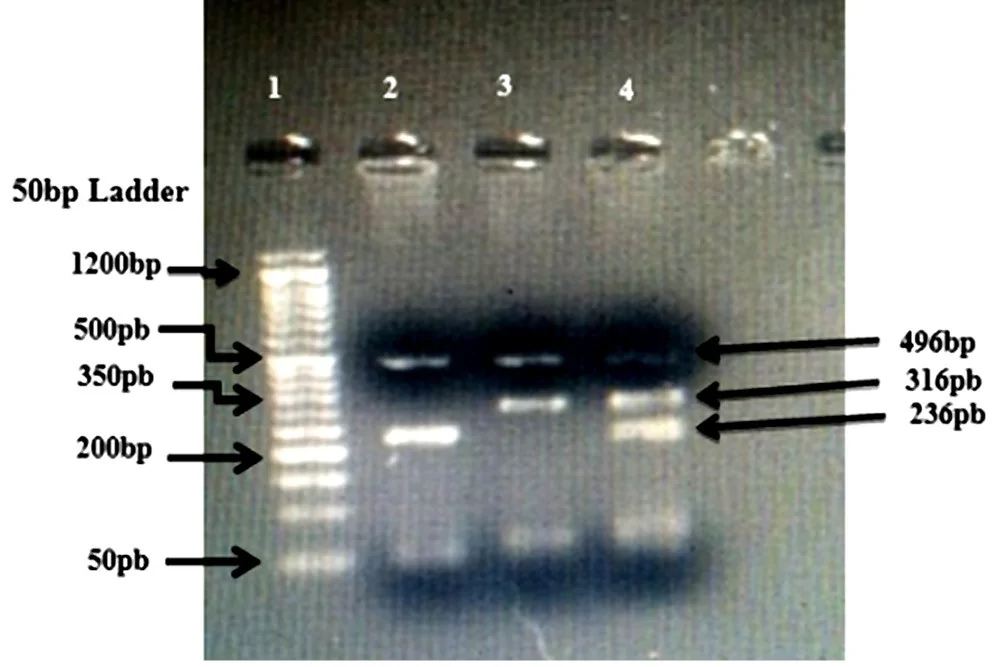
Tetra-ARMS-PCR assay for the genotypes of *CCR3 rs6441961* T > C

 Lane 1 shows a 50 bp DNA ladder, Lane 2 indicates the homozygous TT genotype, Lane 3 indicates the homozygous CC genotype, and Lane 4 indicates the presence of both alleles and generates a heterozygous TC genotype.

###  Statistical Analysis

 All the tests were performed using SPSS software version 20 (IBM Inc. Armonk, NY, USA). Chi-square test helped in assessing the association between *CCR3 rs6441961* T > C polymorphism and celiac disease. It also calculated the genotypic frequencies, while allelic frequencies were calculated by the gene counting method. To further explore the *CCR3 rs6441961* T > C polymorphism associated with CD risk, multinomial regression was applied. Both adjusted (adjusted for age and sex) and unadjusted models were used for *CCR3 rs6441961* T > C genotypes and alleles.

## Results

 Genotypic data followed the Hardy-Weinberg Equilibrium (χ2 = 1.28). Genotypic frequencies demonstrated that the TT genotype (41.8%) was more common in healthy controls, while in the disease group, the prevalence of the TT and TC genotypes was almost the same (37.1% and 37.2%, respectively). Furthermore, the CC genotype was more common in the disease group than in healthy controls (25.8% vs. 18.2%). Intergroup comparison of allelic frequencies showed that the T allele is more prevalent in healthy controls (61.9% vs. 55.8%), while the C allele is more frequently observed in the disease group (44.2% vs. 38.1%).

 However, intragroup comparisons revealed higher frequencies of the T allele in both groups. Although the C allele and CC genotype were predominantly carried by the disease group (as compared with healthy controls), the chi-square test could not establish any association between CD and *CCR3 rs6441961* T > C genotypes (χ2 = 0.74, *P* = 0.69) and alleles (χ2 = 0.66, *P* = 0.44) in the Punjabi Pakistani population (Table 2).

**Table 2:** Genotypic & allelic frequencies for *rs6441961* in the Punjabi population of Pakistan

**CCR3 rs6441961 polymorphism**		**Healthy controls ** **N=55**	**Disease group** **N=35**	**Significance**
Genotypes	TT	23 (41.8%)	13 (37.1%)	χ2 = 0.74 *P* = 0.69
	CC	10 (18.2%)	9 (25.8%)	
	TC	22 (40%)	13 (37.2%)	
Alleles	C	42 (38.1%)	31 (44.2%)	χ2 = 0.66 *P* = 0.44
	T	68 (61.9%)	39 (55.8%)	

 Multinomial regression analysis was applied to further explore the association between CD and *CCR3 rs6441961* T > C polymorphism. Results revealed that *CCR3 rs6441961* CC genotype [OR 1.6 (CI: 0.52-4.9), *P* = 0.42] and TC genotype [OR 1.1 (CI: 0.4-2.8), *P* = 0.93] can increase the risk of CD, however, this association could not reach the significance level (*P* < 0.05, Table 3). Similar results were obtained when the *CCR3 rs6441961* C allele was compared with the T allele [OR 1.3 (CI: 0.7-2.4), *P* = 0.42].

**Table 3:** Multinomial regression table for genotypes and alleles for celiac disease

**Multinomial Regression Analysis[Odds ratio (Confidence interval) ** ***P*** ** value]**			
		**Unadjusted**	**Adjusted**
Genotypes	CC	1.6 (0.52-4.9) 0.42	1.6 (0.51-4.9) 0.44
	TC	1.1 (0.4-2.8) 0.93	0.92 (0.35-2.6) 0.92
	TT	Ref	Ref
Alleles	C	1.3 (0.7-2.4) 0.42	1.3 (0.69-2.3) 0.46
	T	Ref	Ref

 Results remained non-significant even after adjustment for confounding factors like age and sex. Although the *CCR3 rs6441961* TC genotype [OR 0.92 (CI: 0.35-2.6) *P* = 0.92] showed a protective behavior after adjustment for age and sex, this was not statistically significant (Table 3). Thus, it could be deduced that the risk of celiac disease in the Punjabi Pakistani population is independent of the *CCR3 rs6441961* T > C genetic variation.

## Discussion

 This study was designed to explore the association of *CCR3 rs6441961T* > C with CD in Punjabi Pakistani population. Genotyping of the above-mentioned polymorphism was done by using an in-house developed tetra-ARMS-PCR. To the best of our knowledge, the current study is the first study in Pakistan that tried to explore the genetic markers associated with CD; however, most of the previous studies conducted in Pakistan addressed serological markers and symptoms of CD.

 The current study demonstrates that the *CCR3 rs6441961* TC and TT genotypes are predominantly present in healthy controls and the patient group. Although the C allele and CC genotype were more prevalent in patients with CD, this did not reach statistical significance. Results of logistic regression analysis adjusted for confounding factors (age and sex) were also concordant with previous findings and reported no association between *CCR3 rs6441961* T > C polymorphism and risk of CD in the Punjabi Pakistani population.

*CCR3 rs6441961* T > C polymorphism is located 44kb from the 3` end of the *CCR3* gene and overlaps with a pseudogene (Ubiquinol cytochrome c reductase protein 2 pseudogene 1). Although this polymorphism is not located inside the *CCR3* gene, it is a part of a gene cluster in which *CCR3* is the closest gene to the *rs6441961* T > C polymorphism. *CCR3* belongs to a family of G-coupled receptors and responds to several chemokines, including *CCL5, CCL7, CCL11, CCL13*, and *CCL26*. In 2008, Hunt and colleagues, forthefirst time, reported the association of *rs6441961* with the risk of CD.^27^ After this discovery, several studies were conducted to further explore the association of *CCR3 rs6441961* with the risk of CD in different populations.^28^ However, the results of these studies were inconclusive. Most of the genetic association studies were conducted on European populations; however, none of the studies addressed the Asian populations.

 Genome-wide association studies showed that *rs6441961* influences the risk of CD in a population-based manner. Some populations like the North American ^28^, European ^14^, Swedish– Norwegian ^20^, Spanish ^22^ and Dutch ^29^ showed an association between *rs6441961* and CD risk. While the Italian population could not establish a link between *rs6441961* and CD risk.^30^ Another replicative study on a Spanish cohort consisting of 1094 patients with CD and 540 controls showed no association for *CCR3 rs6441961*, which further complicated the interaction of said polymorphism with risk of CD and concluded that the discordant findings on association of *rs6441961* with CD could be a consequence of arbitrary candidate gene selection within association signals that are not based on functional studies.^31^

 Some researchers highlighted the similarities in risk loci and disease pathogenesis of autoimmune diseases like CD, type 1 diabetes, rheumatoid arthritis, and collagenous sprue.^32^ Immune factors play an important role in the pathobiology of these diseases. *CCR3* is also an important component of the immune system. Smyth and others showed that the *CCR3 rs6441961* polymorphism located on 3q21 is shared by type 1 diabetes and CD ^19^, however, *rs6441961* does not confer the risk of rheumatoid arthritis (which is also an autoimmune inflammatory disease).^29^

 As stated earlier, the association of *rs6441961* with CD has been widely studied only in European populations, yielding inconclusive results owing to differences in genetic composition. Thus, there is a dire need to study the non-HLA genetic loci in Asian populations to explore the genetic markers that increase the risk of CD. The current study is the first genetic association study conducted on the Pakistani population, and it paves the way for upcoming large-scale studies to elucidate the genetic risk factors for CD in Asian populations.

## Conclusion

 In the current study, a rapid, specific, and cost-effective tetra-ARMS-PCR genotyping assay was developed for genotyping of the *CCR3 rs6441961* T > C polymorphism, which was hypothesized to increase the risk of CD. However, statistical analysis revealed that the risk of CD in the Punjabi Pakistani population is independent of the *CCR3 rs6441961* T > C polymorphism. The small sample size and higher frequency of the CC genotype in the selected sample cohort hinder the generalizability of results and emphasize the need to conduct validation studies with a larger sample size to confirm this association.
